# Brazing of TC4 Alloy Using Ti-Zr-Ni-Cu-Sn Amorphous Braze Fillers

**DOI:** 10.3390/ma17153745

**Published:** 2024-07-29

**Authors:** Zhan Sun, Boyu Zhang, Degang Li, Xinxin Zhu, Qing Chang, Bo Zhang, Lixia Zhang, Weimin Long, Sujuan Zhong

**Affiliations:** 1 State Key Laboratory of Precision Welding & Joining of Materials and Structures, Harbin 150001, China; hitsunzhan@outlook.com (Z.S.); 524201736@163.com (B.Z.); 17862702932@163.com (D.L.); 15266847935@163.com (X.Z.); cty1990@hotmail.com (Q.C.); zburke@126.com (B.Z.); 2 State Key Laboratory of Advanced Brazing Filler Metals & Technology, Zhengzhou Research Institute of Mechanical Engineering Co., Ltd., Zhengzhou 450001, China; longweimin@camsouth.com.cn (W.L.); 13937109560@163.com (S.Z.)

**Keywords:** TC4, Ti-based braze filler, vacuum brazing, microstructure

## Abstract

In order to address the issues of excessive brittle intermetallic compounds (IMC) formation in the TC4 brazed joints, two types of novel Ti-Zr-Cu-Ni-Sn amorphous braze fillers were designed. The microstructure and shear strength of the TC4/Ti-Zr-Ni-Cu-Sn/TC4 brazed joints were studied by scanning electron microscopy (SEM), transmission electron microscopy (TEM), X-ray diffractometer (XRD) and electronic universal materials testing machine. The results show that the optimized Ti_35_Zr_25_Ni_15_Cu_20_Sn_5_ braze filler whose chemical composition is closer to the eutectic point possesses a lower melting point compared with the equiatomic Ti_23.75_Zr_23.75_Ni_23.75_Cu_23.75_Sn_5_. This was beneficial to the sufficient diffusion of Cu and Ni elements with the base metal during brazing and reduces the residual (Ti,Zr)_2_(Ni,Cu) content in the joint, which helps to improve the joint performance. The room-temperature and high-temperature shear strength of the TC4 brazed joints using the near eutectic component Ti_35_Zr_25_Ni_15_Cu_20_Sn_5_ filler reached a maximum of 472 MPa and 389 MPa at 970 °C/10 min, which was 66% and 48% higher than that of the TC4 joints brazed with the equiatomic Ti_23.75_Zr_23.75_Ni_23.75_Cu_23.75_Sn_5_ braze filler. Microstructural evolution and the corresponding mechanical response were in-depth discussed.

## 1. Introduction

TC4 is one of the most important titanium alloys used in industrial production. It is widely used in aerospace, aviation and transportation fields due to its low density, good strength and corrosion resistance [1,2,3,4,5]. In order to fully exploit excellent properties of TC4, the structure of components is becoming more complicated. Currently, brazing is the preferred solution for joining complex components since its low joining temperature and little impact on the base metal alloy. Ti-based filler is one of the most commonly used fillers in the titanium alloy brazing process [6]. The joint obtained by Ti-based filler has high mechanical properties and good corrosion resistance. Jing et al. [7]. designed a TiZrCuNi brazing material and successfully brazed TA15 to obtain a joint with good metallurgical bonding. Liu et al. [8] also successfully vacuum-brazed TC4 using a Ti-37.5Zr-10Ni-15Cu (wt%) brazing material. However, the central part of their joints contains a large amount of (Ti,Zr)_2_(Ni,Cu). The microhardness and modulus of (Ti,Zr)_2_(Ni,Cu) did not match the matrix, which greatly limited the joint’s room-temperature and high-temperature shear strength [9]. The main reason for this phenomenon is that the melting point of Ti is high (~1670 °C). Ni and Cu elements are essential elements added into the Ti-based filler, aiming at reducing the melting point of the filler metal. However, this will inevitably lead to the formation of residual brittle (Ti,Zr)_2_(Ni,Cu) intermetallic compounds (IMC) in the central joint [10]. Therefore, the development of new filler metals with optimized chemical composition designed for TC4 brazing is of great importance to achieve robust TC4 brazed joints. Pang et al. [11] used Sn instead of Cu and Ni to design a novel TiZr-based filler metal with a low total content of Cu and Ni and low liquidus temperature. Zhang et al. [12,13] designed a Ti-Zr-Cu-Sn brazing filler to braze TC4 titanium alloy successfully. The results showed that adding Sn to the Ti-based filler metal could effectively reduce the melting point of the filler metal and improve the strength of the joint. However, excessive Sn was easy to react with Zr to produce brittle Zr_5_Sn_3_. Therefore, the content of Sn in brazing metal was usually less than 5 at.% [13].

In this research, two types of Ti-based amorphous braze fillers were proposed, i.e., a Ti_23.75_Zr_23.75_Ni_23.75_Cu_23.75_Sn_5_ braze filler with equiatomic ratio, which complies with high-entropy components and a Ti_35_Zr_25_Ni_15_Cu_20_Sn_5_ braze filler whose chemical composition is much closer to the eutectic point. Microstructural evolution as well as the mechanical response of the TC4 joints brazed with these two braze fillers were investigated and compared. The key factors improving joint strength were further discussed.

## 2. Materials and Methods

Zr and Ti belong to the same group of elements, IVB, and have similar properties. They can dissolve each other indefinitely and Zr can reduce the melting point of the filler metal. Additionally, Ni and Cu can form low-melting-point eutectics with Ti, effectively reducing the melting point of the filler metal, and were often added to Ti-based filler metals [14,15]. Ni and Cu have similar properties and can dissolve each other indefinitely, which can promote the formation of solid solutions. The melting point of Sn is only 231.89 °C and adding it to the filler metal is an effective way to lower the melting point [16]. However, Zr and Sn have a negative mixing enthalpy. To prevent the formation of intermetallic compounds between Zr and Sn, the Sn content is designed to be 5 at.%.

**Figure 1:**
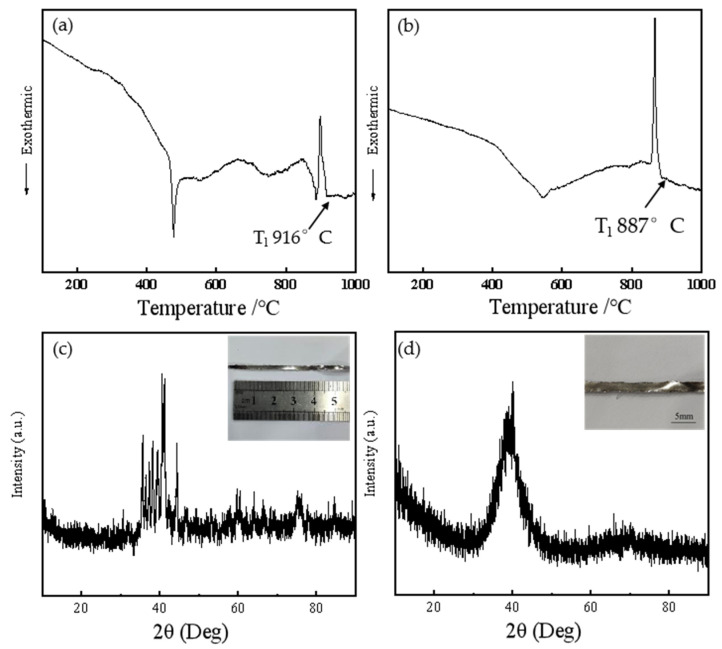
The DSC curve (**a**) and XRD pattern (**c**) of Ti_23.75_Zr_23.75_Ni_23.75_Cu_23.75_Sn_5_ amorphous high-entropy filler ribbons; The DSC curve (**b**) and XRD pattern (**d**) of Ti_35_Zr_25_Ni_15_Cu_20_Sn_5_ amorphous high-entropy filler ribbons.

In this experiment, Ti, Zr, Ni, Cu, and Sn metal particles (ZhongNuo Advanced Material Technology Co., Ltd., Beijing, China) with a purity of 99.99 wt% were used. According to the composition design of the filler, the metal particles of Ti, Zr, Ni, Cu and Sn were weighed. The metal particles were melted into an ingot using a high-vacuum arc furnace (Hefei Kejing Materials Technology Co., Ltd., Hefei, China). Finally, after induction melting in a quartz tube under the high purity argon protection, the ingots were rapidly solidified at a circumferential speed of 20–25 m/s by using single-roll melt spinning technology of a rotating Cu wheel. The resulting filler metal was flexible and had a ribbon shape of 3–4 mm in width and about 50 μm in thickness. In order to determine the melting temperature of amorphous filler metal, the melting characteristics of the brazing alloy were measured by differential scanning calorimetry (DSC) at a heating rate of 10 °C/min. An X-ray diffractometer (XRD) was used to identify the phases in the joint. Cu Kα was selected as the source of X-rays with a scan range of 10–90° and a speed of 5°/min. The results are shown in Figure 1. From the figure, it can be seen that the filler metal ribbon only exhibits a broad, diffuse peak at around 40°, and there were no sharp diffraction peaks throughout the curve. The diffraction peak matches the typical characteristics of amorphous metal, indicating that the filler metal produced was indeed in an amorphous state. This experiment uses TC4 as the base metal, which was cut into two rectangular shapes, 3 mm × 3 mm × 3 mm and 15 mm × 10 mm × 3 mm, using electrical discharge cutting. The samples for microstructure analysis and shear strength testing were lap joint assembly models. Impurities such as oxides on the surface of the alloy must be removed before brazing. The surface of the substrate joint was ground with 800# silicon carbide sandpaper and polished with a diamond polishing agent. Then, it was ultrasonically cleaned in anhydrous ethanol for 300 s and dried in air.

During brazing, the samples were assembled in the order of TC4/Ti-Zr-Cu-Ni-Sn/TC4, and the assembled components were placed in a vacuum brazing furnace (Shenyang Xinlantian Vacuum Technology Co., Ltd., Shenyang, China) for brazing experiments. In order to study the microstructure and properties of the joints under different brazing conditions, the brazing temperature was set in the range of 930–990 °C. During brazing, the temperature was heated from room temperature to 400 °C at a rate of 15 °C/min, and then further heated from 400 °C to 800 °C at a rate of 10 °C/min. Finally, the heating temperature was raised from 800 °C to the required brazing temperature and held at this temperature at a rate of 5 °C/min. The cooling rate was 5 °C/min.

**Figure 2:**
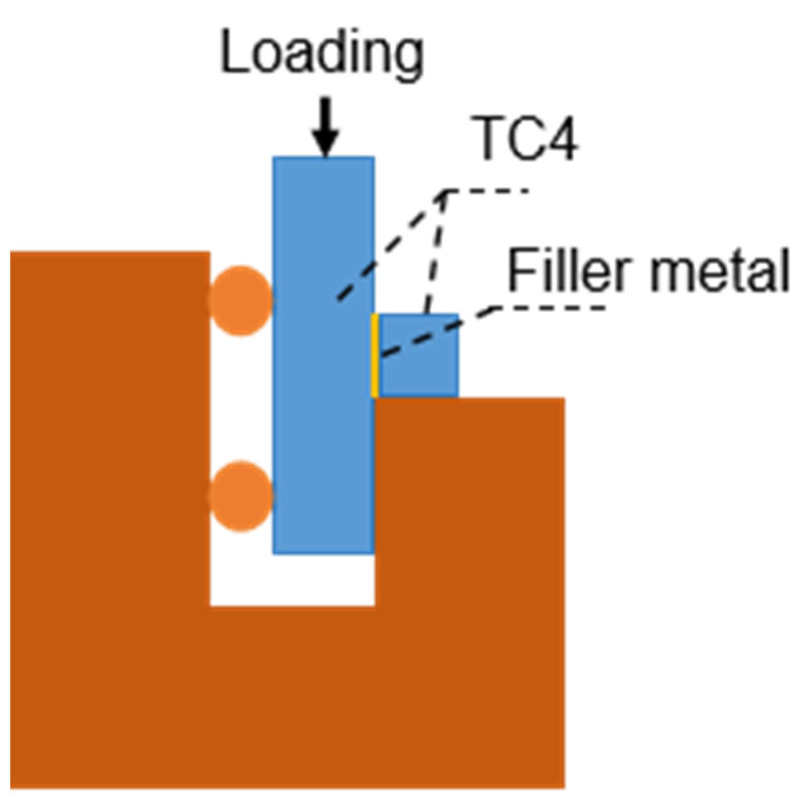
Diagram of the shearing test.

To determine the phases of the brazed joint, scanning electron microscopy (SEM) was used to analyze the microstructure of the joint with a field emission gun operated at 20 kV, and energy dispersive spectroscopy (EDS) was used to identify the elements present in each phase. Transmission electron microscopy (TEM, at 200 kV) was used to observe the phases at the nanoscale level. To determine the shear strength of the joint, an electronic universal testing machine (AG-x Plus) was used to perform shear tests at a speed of 0.5 mm/min, and the shear method is shown in Figure 2. To avoid its influence on the shear strength of the joint, The fillet of the joint should be removed before the shear test. To ensure the accuracy and authenticity of the shear strength data, the shear strength values of the joint were the average of five samples.

## 3. Results and Discussion

### 3.1. Vacuum Brazing of TC4 with Equiatomic Designed Ti_23.75_Zr_23.75_Ni_23.75_Cu_23.75_Sn_5_ Filler Metal

**Figure 3:**
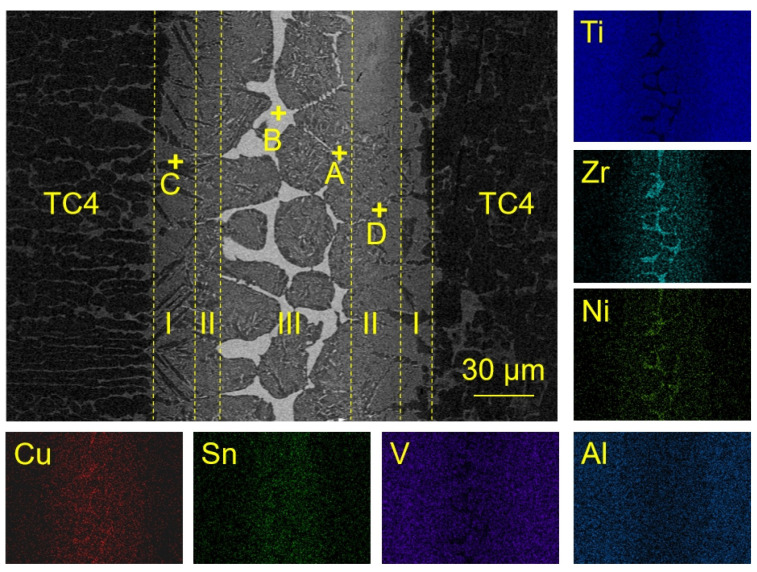
The microstructure of brazed joints using Ti_23.75_Zr_23.75_Ni_23.75_Cu_23.75_Sn_5_ and element distribution map.

Based on the differential scanning calorimetry (DSC) analysis, the liquidus temperature of Ti_23.75_Zr_23.75_Ni_23.75_Cu_23.75_Sn_5_ filler metal was approximately 916 °C. The microstructure of the joint under the condition of 930 °C/10 min was analyzed. The microstructure and chemical composition of the joint are shown in Figure 3. According to the morphology of the microstructure, the joint can be divided into 3 regions; Region I, where the dark grey phase C grew in a needle shape towards the center of the brazed seam; Region II, where the light grey phase D occupied the majority of the area; and Region III, where the residual filler metal layer remained in the central part. The formation of Region III indicated that 930 °C/10 min was not strong enough, as it resulted in insufficient diffusion and reactions between the filler metal and the base metal. The elemental mapping images indicated that a certain degree of mutual diffusion occurred, while there were Ni and Zr-rich areas formed in the central part of the joints. From the interfacial microstructure shown in Figure 3, the dark gray Phase C has the same contrast as the dark gray phase in the base metal. According to the EDS results in Table 1, Phase C contains a large amount of Al and low amounts of Ni and Cu. Since Al was a strong α-Ti stabilizer element, it was speculated that the dark gray phase C was α-Ti. Al and Ti from the base metal continuously dissolved into the liquid brazed seam and the α-Ti probably formed in the process of isothermal solidification when holding the temperature [10].

Phase D contained a two-phase microstructure with different contrasts and small sizes, which is the typical feature of eutectoid microstructure. For the chemical composition, there is no dendrite in the D phase, which is completely composed of a fine eutectoid structure. Phase D mainly contains a large amount of Al, Ni and Cu. The dark gray phase which was similar to Phase C was speculated to be α-Ti. The binding energy of Cu and Ni elements with Ti (−11.72 kJ/mol [17] and −33.24 kJ/mol [18]) was high, and it was easy to form intermetallic compounds (Ti,Zr)_2_(Ni,Cu) with Ti. According to the analysis of XRD results, the light gray phase was confirmed to be (Ti,Zr)_2_(Ni,Cu). Therefore, Phase D was presumed to be a eutectoid microstructure of α-Ti + (Ti,Zr)_2_(Ni,Cu) [19]. Phase A contained high contents of Zr and Sn. When combined with the XRD pattern of the brazed joint, Phase A was speculated to be Zr_5_Sn_3_.

**Figure 4:**
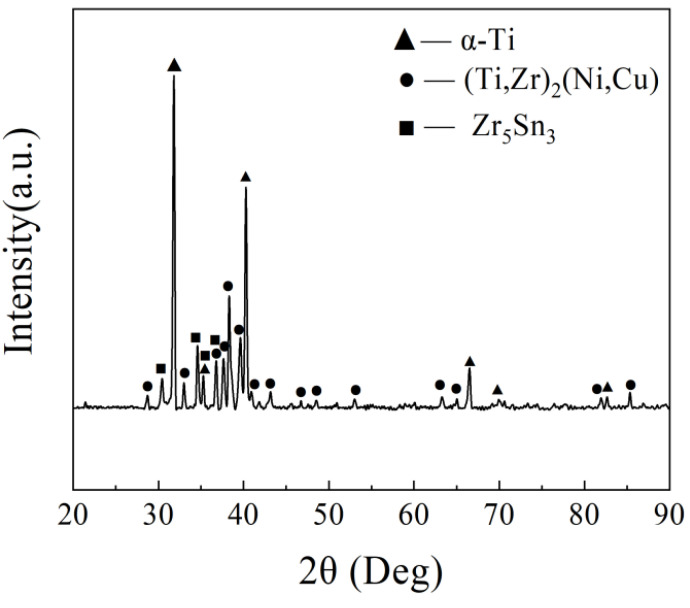
XRD pattern of brazed joints using Ti_23.75_Zr_23.75_Ni_23.75_Cu_23.75_Sn_5_ at 930 °C/10 min.

Phase B located in the center of the joint mainly contained four elements: Ti, Zr, Ni, and Cu. However, Ti content in this phase was much lower than that in Phase C and D. Combined with Table 1 and Figure 4, it was speculated that Phase B was a (Ti,Zr)_2_(Cu,Ni) intermetallic compound. The reason for its formation was that during the brazing process, a large amount of Ti continually dissolves and diffuses from the base metal into the central joint. As the distance increases, the diffusion of Ti also decreases. At the same time, Cu and Ni elements in the center of the joint were relatively difficult to completely diffuse toward the TC4 base alloy. Therefore, the braze filler in the central joint can hardly be enriched by the dissolved Ti, leading to a higher content of Cu and Ni in this area. Therefore, a large amount of (Ti,Zr)_2_(Cu,Ni) forms as a result in the center of the joint. The formation of Phase B shows strong negative effects on the mechanical strength of the TC4 brazed joints [20], which should be carefully controlled.

Based on the above analysis, when the brazing parameter was 930 °C/10 min, the microstructure of the TC4 joint brazed with the equiatomic Ti_23.75_Zr_23.75_Ni_23.75_Cu_23.75_Sn_5_ mainly consisted of acicular α-Ti, α-Ti + (Ti,Zr)_2_(Ni,Cu) eutectoid microstructure. It also showed a continuous layer of residual (Ti,Zr)_2_(Ni,Cu) intermetallic compounds and a small amount of Zr_5_Sn_3_ intermetallic compounds in the joint center.

### 3.2. Vacuum Brazing of TC4 with Near-Eutectic Ti_35_Zr_25_Ni_15_Cu_20_Sn_5_ Filler Metal

In the process of brazing TC4 with equiatomic Ti_23.75_Zr_23.75_Ni_23.75_Cu_23.75_Sn_5_, there were still large brittle (TiZr)_2_(CuNi) intermetallic compounds in the joint. This may be strongly related to the high content of Cu and Ni in the brazing filler. At the same time, Cu and Ni were essential melting point depressants in the Ti-based braze filler, which should not be totally eliminated. Since Ti-Zr and Cu-Ni were pairs of elements with similar properties and were infinitely soluble with each other, they can be simplified into two elements, Zr and Ni. According to the Zr-Ni phase diagram, the atomic ratio of Zr-Ni eutectic point was 63:37. Therefore, by controlling the total atomic ratio of Ti-Zr and Cu-Ni around this proportion, it should be closer to the eutectic point of the multicomponent alloy. Therefore, the filler metal was designed as Ti_35_Zr_25_Ni_15_Cu_20_Sn_5_. Based on the DSC analysis, the liquidus temperature of Ti_35_Zr_25_Ni_15_Cu_20_Sn_5_ filler metal was approximately 887 °C, which was about 29 °C lower than that of Ti_23.75_Zr_23.75_Ni_23.75_Cu_23.75_Sn_5_. This means that the chemical composition of Ti_35_Zr_25_Ni_15_Cu_20_Sn_5_ filler was closer to the eutectic point.

**Figure 5:**
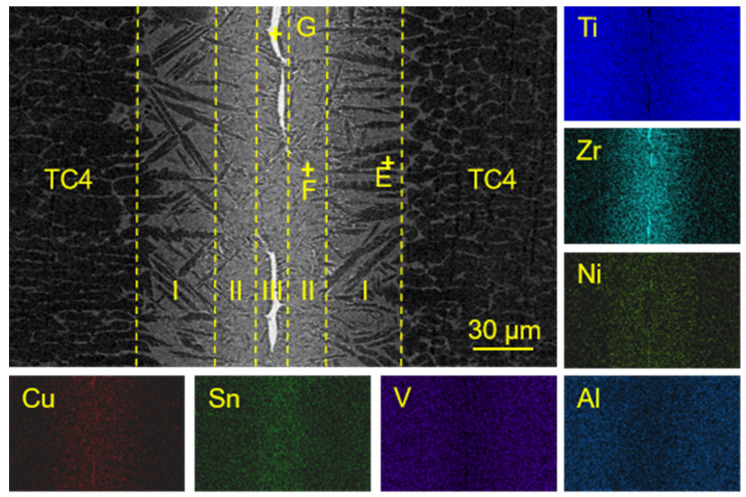
The microstructure of brazed joints using Ti_35_Zr_25_Ni_15_Cu_20_Sn_5_ at a brazing temperature of 930 °C for 10 min, and the corresponding element distribution map.

**Figure 6:**
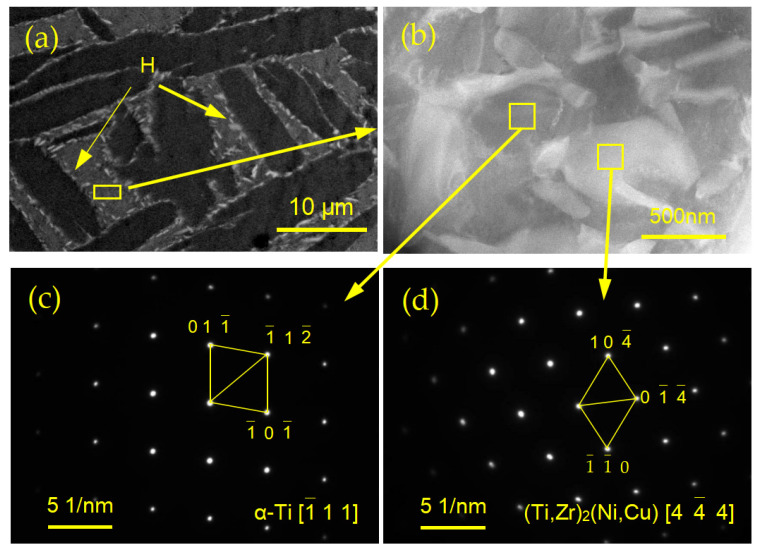
(**a**) Enlarged view of eutectoid microstructure; (**b**) the FIB images, (**c**,**d**) SAEDPs of the gray eutectoid microstructure.

The microstructure of the TC4 joint brazed with Ti_35_Zr_25_Ni_15_Cu_20_Sn_5_ amorphous high-entropy brazing filler metal at 930 °C/10 min is shown in Figure 5. EDS results of the joint are shown in Table S1, and the XRD pattern is shown in Figure S1. According to the different morphologies, the microstructure of the brazed seam can also be roughly divided into three regions, which was similar to the joint brazed using equiatomic Ti_23.75_Zr_23.75_Ni_23.75_Cu_23.75_Sn_5_. However, Region I was much larger and Region III was smaller. Region I composed of α-Ti dendrites and tiny eutectoid microstructure plays a strong positive effect on joint strength. In order to further determine the tiny eutectoid microstructure of joints, transmission electron microscopy (TEM) was used to analyze the eutectic microstructure. Figure 6b was a FIB (Focused Ion beam) sample, and Figure 6c,d were SAEDPs (Selected area electron diffraction) of the two phases in the eutectoid microstructure. According to the diffraction patterns, the darker phase in the figure with a hexagonal lattice structure was the α-Ti phase, while the lighter phase was the (TiZr)_2_(NiCu) phase. As shown at H in Figure 6a, it can be seen that white tiny (Ti,Zr)_2_(Ni,Cu) phases are distributed around the dark α-Ti phase. Therefore, the gray phase in Regions I and II was indeed the eutectoid microstructure of α-Ti and (TiZr)_2_(NiCu).

**Figure 7:**
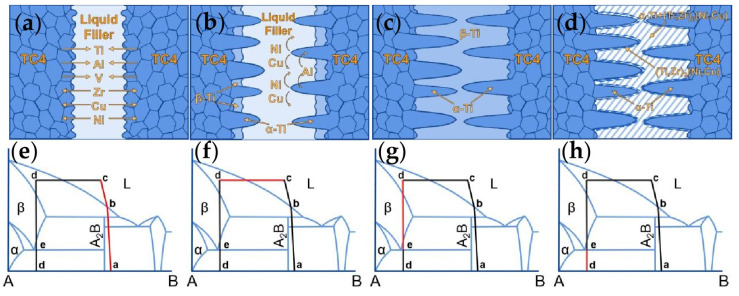
Microstructure evolution and diffusion behavior during the brazing process (**a**,**e**) Melting of the Ti_35_Zr_25_Ni_15_Cu_20_Sn_5_ braze filler; (**b**,**f**) β-Ti and α-Ti formation during the isothermal solidification process; (**c**,**g**) The liquid filler metal solidified to form β-Ti; (**d**,**h**) Eutectoid phase transition from β-Ti to α-Ti + (Ti,Zr)_2_(Ni,Cu).

Figure 7 shows the microstructural evolution and elemental diffusion behavior of the TC4 brazed joints. Due to the complexity of the phase diagram of the multi-component alloy, it is not conducive to the detailed analysis of the microstructure evolution of the brazing. Since the phase diagrams of Ti-Cu, Ti-Ni, Zr-Cu and Zr-Ni are very similar, we merged and simplified the similar elements in the fillers. A was used to represent Ti and Zr elements, and B was used to represent Cu and Ni elements. The simplified phase diagram is shown in Figure 7e–h. During the brazing process, the heating temperature rose first until it reached the liquid-phase region of the Ti-Zr-Ni-Cu-Sn braze filler (indicated by the red line in Figure 7e), resulting in the melting of the braze filler (shown in Figure 7a). When further extending the heating duration, Ti from the base metal continuously dissolved into the filler. This made the brazing seam wider, and the composition of the braze filler near the base metal changed correspondingly. When the chemical composition of the liquid braze filler/TC4 alloy interface moved from the liquid-phase region into the solid-liquid two-phase region (indicated by the red line in Figure 7f), β-Ti started to form here (shown in Figure 7b). In addition, while the base metal dissolved, Al from the base metal also entered the filler. Since Al is a stable element of the α-Ti phase and a small amount of Al can also increase the melting point of titanium alloy [21]. Constitutional supercooling happened at the liquid braze filler/solid TC4 alloy interface. Which resulted in the continuous growth of the needle-like α-Ti towards the center of the joints [22] (shown in Figure 7b). Besides, due to the low solubility of Cu and Ni in α-Ti, Cu and Ni will be discharged and enriched in the solid-liquid front during the dendrite growth of α-Ti as shown in Figure 7b. When the holding stage ended, the heating temperature decreased into the solid-phase region indicated by the red line as shown in Figure 7g, which means, in this process, the residual liquid filler metal solidified into β-Ti as shown in Figure 7c. When the temperature further decreased, it would reach the two-phase regions indicated by the red line shown in Figure 7h. The eutectoid transition from β-Ti to α-Ti + (Ti,Zr)_2_(Ni,Cu) occurred. Due to the high Cu and Ni content near the α-Ti dendrites, it was easier to form (Ti,Zr)_2_(Ni,Cu). Thus, the continuously distributed (Ti,Zr)_2_(Ni,Cu) formed near the α-Ti dendrites as shown in Figure 7d.

Since Cu and Ni were both melting-reducing elements, enrichment at the solid-liquid front will reduce the melting point of the filler here, thereby inhibiting the growth of α-Ti. Compared with Ti_23.75_Zr_23.75_Ni_23.75_Cu_23.75_Sn_5_, the content of Cu and Ni elements in Ti_35_Zr_25_Ni_15_Cu_20_Sn_5_ filler was less, which has a weak inhibitory effect on the growth of α-Ti. Therefore, the size of α-Ti in the joint brazed with Ti_35_Zr_25_Ni_15_Cu_20_Sn_5_ was larger, and the area of Region I also became wider.

Due to the close-packed atomic structure of α-Ti, the diffusion coefficients of Cu and Ni were low [23]. Due to the obstruction of α-Ti in Region I, the diffusion rate of Cu and Ni elements in the center to the base metal slowed down. In addition, Region III was far away from the base metal, and it was difficult to obtain Ti dissolved from the base metal to dilute the content of Cu and Ni. As a result, a continuous (Ti,Zr)_2_(Ni,Cu) was formed in the center with high Cu and Ni content. Compared with Ti_23.75_Zr_23.75_Ni_23.75_Cu_23.75_Sn_5_, the content of Cu and Ni elements in Ti_35_Zr_25_Ni_15_Cu_20_Sn_5_ filler was less. Therefore, the enrichment of Cu and Ni in the center was not strong. The continuous (Ti,Zr)_2_(Ni,Cu) brittle phase was also less, and the area of Region III was smaller.

### 3.3. Effect of Brazing Temperatures on Microstructure and Mechanical Properties of the TC4 Joints Brazed with Ti_35_Zr_25_Ni_15_Cu_20_Sn_5_

**Figure 8:**
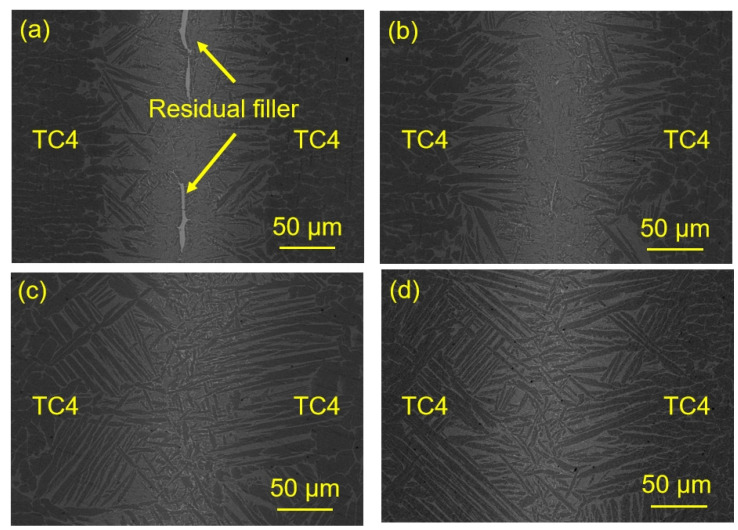
The microstructure of the joints brazed with Ti_35_Zr_25_Ni_15_Cu_20_Sn_5_ at various temperatures for 10 min (**a**) 930 °C; (**b**) 950 °C; (**c**) 970 °C; (**d**) 990 °C.

Figure 8 shows the effects of the brazing temperatures on the microstructure evolution of the TC4/Ti_35_Zr_25_Ni_15_Cu_20_Sn_5_/TC4 joint. It can be seen from the figure that the width of the brazing seam increases with the increase of brazing temperature. This indicates that the reaction between the base metal and the filler was intensified, and more Ti, Al and V elements dissolved into the filler from the base metal. This makes the Cu and Ni elements in the center of the joint more fully diluted. At the same time, the increase of elements dissolved in base metal to filler metal was also conducive to the growth of α-Ti. Therefore, with the increase in temperature, the size of α-Ti in the joint increases gradually.

When the brazing temperature was 930 °C, there was a small amount of (Ti,Zr)_2_(Ni,Cu) intermetallic compounds remained in the center of the joint. The continuous (Ti,Zr)_2_(Ni,Cu) phases in the center disappear when the brazing temperature reaches 950 °C. According to Fick’s law, as the brazing temperature increased, the diffusion rate of base metal elements into the brazing seam increased. Therefore the reaction between the base metal and the filler was intensified, and more Ti, Al and V elements dissolved into the filler from the base metal, which made Cu and Ni in the central joint more fully diluted. The residual brittle (Ti,Zr)_2_(Ni,Cu) phases were thus suppressed. When the brazing temperature reaches 970 °C, Region II and III disappear, and the joint is completely composed of α-Ti and α-Ti + (Ti,Zr)_2_(Ni,Cu) eutectoid microstructure. This was mainly due to the increase in temperature, the dissolution rate of the elements from the base metal to the filler was accelerated. These elements from the base metal and high-temperature both accelerated the growth of α-Ti. When the α-Ti grew from both sides of the base metal contact with each other, Region II disappeared. When the temperature reaches 990 °C, the joint microstructure is similar to that at 970 °C. However, the microstructure of the base material has changed. This was because when the brazing temperature reached 990 °C, the brazing temperature was already higher than the phase transformation temperature of the base metal, resulting in changes in the microstructure of the TC4 base metal [24,25].

**Figure 9:**
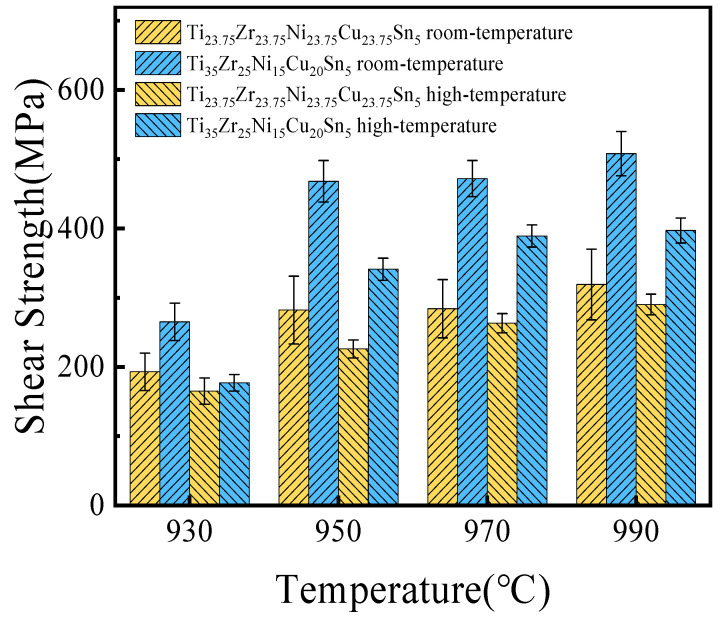
Average shear strength of joints at different brazing temperatures for 10 min.

Figure 9 shows the room-temperature and high-temperature (450 °C) shear strength of the joints brazed with two high-entropy brazing alloys at various brazing temperatures for 10 min. At the same time, it can also be seen from the figure that the variation trend of shear strength at room temperature and high temperature (450 °C) was similar, and it both increased at first with the elevation of holding temperature. Under the same brazing time, the room-temperature and high-temperature shear strength of the joint brazed with Ti_35_Zr_25_Ni_15_Cu_20_Sn_5_ was greater than that of the joint brazed with Ti_23.75_Zr_23.75_Ni_23.75_Cu_23.75_Sn_5_ at various brazing temperatures.

The room-temperature shear strength of the joint brazed with Ti_35_Zr_25_Ni_15_Cu_20_Sn_5_ increased with the increase in temperature. However, when the brazing temperature was 950 °C, compared with 930 °C, the joint room-temperature strength increased the most (increased by 203 MPa). Combined with Figure 8a,b, it can be seen that the continuous (Ti,Zr)_2_(Ni,Cu) phase in the joint disappears when the brazing temperature is 950 °C. That may be the reason for the substantial increase in the shear strength of the joint. This also indicates that continuous (Ti,Zr)_2_(Ni,Cu) was the key factor limiting the joint strength. When the brazing temperature reaches 970 °C, the size of the α-Ti phase in the joint increases. α-Ti solid solution has a certain toughness and good mechanical properties. Therefore, the joint strength was further improved with the increase of α-Ti size. At the same time, the joint strength reaches the maximum when the brazing temperature reaches 990 °C. However, at 990 °C, TC4 undergoes α-Ti⟶β-Ti phase transformation, and the structural properties of the base metal have changed. Therefore, in this experiment, the best brazing parameters should be 970 °C/10 min. When the brazing parameter was 970 °C/10 min, the room-temperature and high-temperature shear strength of the TC4 joint brazed with Ti_35_Zr_25_Ni_15_Cu_20_Sn_5_ was 472 MPa and 389 MPa. Compared with the joint brazed with the traditional equiatomic high-entropy brazing alloy, which has a room-temperature and high-temperature shear strength of 284 MPa and 263 MPa, the joint strength increased by about 66% and 48%. The high-temperature shear strength of the joint brazed with Ti_35_Zr_25_Ni_15_Cu_20_Sn_5_ had reached 82% of its room-temperature shear strength. It showed that the joint brazed with Ti_35_Zr_25_Ni_15_Cu_20_Sn_5_ had good high-temperature properties.

**Figure 10:**
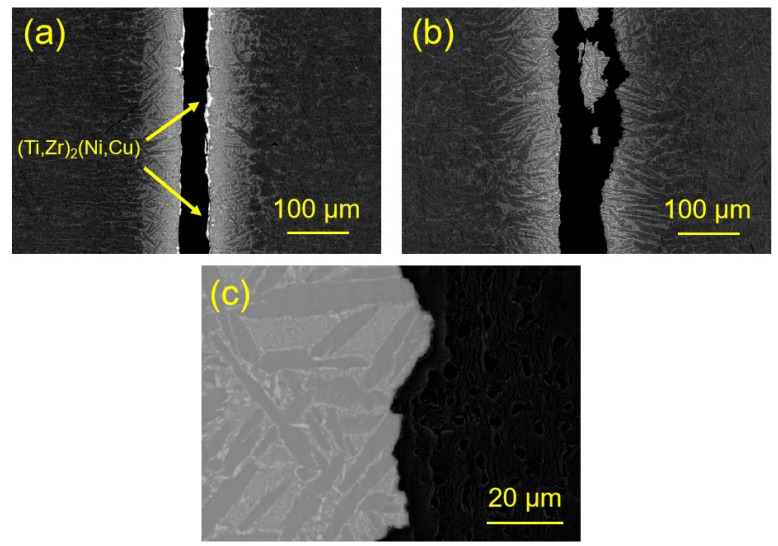
Fracture path of the joints brazed with Ti_23.75_Zr_23.75_Ni_23.75_Cu_23.75_Sn_5_. (**a**) 930 °C/10 min; (**b**) 970 °C/10 min; (**c**) The local enlargement of fracture path in (**b**).

The fracture path of the Ti_35_Zr_25_Ni_15_Cu_20_Sn_5_ brazing joint brazed at different temperatures is shown in Figure 10. It can be seen from the figure that the fracture of the brazing joint at both brazing temperatures was located in the center of the joint. However, there was white residual (Ti,Zr)_2_(Ni,Cu) brittle phase at the fracture morphology of the joint brazed at brazing temperature of 930 °C, which indicates that (Ti,Zr)_2_(Ni,Cu) was a weak phase in the brazing seam organization, greatly weakening the shear strength of the joint. In contrast, the fracture morphology of the joint brazed at 970 °C does not show a large amount of (Ti,Zr)_2_(Ni,Cu) brittle phase. The fracture path was locally amplified, as shown in Figure 10c. It can be seen from the figure that the fracture mostly occurs inside the shallow gray phase α-Ti + (Ti,Zr)_2_(Ni,Cu), and the crack grows along the α-Ti/α-Ti + (Ti,Zr)_2_(Ni,Cu) interface. The reason why the joint presents this fracture mode was that there was a significant difference in mechanical properties between (Ti,Zr)_2_(Ni,Cu) and α-Ti, and the stress concentration was easy to occur at the interface [26]. Cracks were prone to initiate and propagate here, ultimately leading to joint fracture.

## 4. Conclusions

In this study, novel equiatomic Ti_23.75_Zr_23.75_Ni_23.75_Cu_23.75_Sn_5_ and near-eutectic Ti_35_Zr_25_Ni_15_Cu_20_Sn_5_ high-entropy braze fillers were proposed and successfully used to braze a TC4 alloy. Several conclusions were summarized as follows:

1. The microstructure of the TC4/Ti_35_Zr_25_Ni_15_Cu_20_Sn_5_/TC4 joints brazed at 930 °C/10 min mainly consists of 3 regions; Region I, composed of needle-like α-Ti and α-Ti + (Ti,Zr)_2_(Ni,Cu) eutectoid microstructure; Region II, composed of totally α-Ti + (Ti,Zr)_2_(Ni,Cu) eutectoid microstructure; and Region III, composed of residual (Ti,Zr)_2_(Ni,Cu) phase. The content of the residual (Ti,Zr)_2_(Ni,Cu) was much lower after using the near-eutectic Ti_35_Zr_25_Ni_15_Cu_20_Sn_5_ braze filler.
2. Increasing the brazing temperature from 930 °C to 950 °C can effectively eliminate the continuous residual brittle (Ti,Zr)_2_(Ni,Cu) phases. When the temperature increased to 970 °C, the size of α-Ti in the joint increased, and the Region II with total eutectoid microstructure of α-Ti + (Ti,Zr)_2_(Ni,Cu) disappeared. The maximum room-temperature and high-temperature shear strength reached 472 MPa and 389 MPa. The elimination of brittle residual (Ti,Zr)_2_(Ni,Cu) phases and enrichment of needle-like α-Ti phases together contributed to robust TC4 brazed joints.
3. The continuous distribution of residual brittle (Ti,Zr)_2_(Ni,Cu) phase in the central joints at low brazing temperatures was the key factor limiting the shear strength of the TC4 brazed joints. When the large-size brittle (Ti,Zr)_2_(Ni,Cu) phase disappeared, the fracture changed from the brittle (Ti,Zr)_2_(Ni,Cu) phase to the tiny eutectic α-Ti + (Ti,Zr)_2_(Ni,Cu) phase.

## Figures and Tables

**Table 1:** Element content (at.%) of each phase of the joint using Ti_23.75_Zr_23.75_Ni_23.75_Cu_23.75_Sn_5_ at 930 °C/10 min.

Element	Ti	Al	V	Zr	Ni	Cu	Sn	Possible Phase
A	19.08	2.90	0.82	46.20	1.22	1.50	28.28	Zr_5_Sn_3_
B	39.70	9.23	1.34	20.83	14.32	14.12	0.46	(Ti,Zr)_2_(Ni,Cu)
C	86.94	11.31	1.53	0.04	0.09	0.07	0.02	α-Ti
D	60.91	8.53	2.61	11.03	8.33	7.68	0.91	α-Ti + (Ti,Zr)_2_(Ni,Cu)

## Data Availability

The original contributions presented in the study are included in the article, further inquiries can be directed to the corresponding author.

## Supplementary Materials

The following supporting information can be downloaded at https://www.mdpi.com/article/10.3390/ma17153745/s1, Table S1: Element content (at.%) of each phase of the joint using Ti_35_Zr_25_Ni_15_Cu_20_Sn_5_ at 930 °C/10 min, Figure S1: The XRD pattern of brazed joints using Ti_35_Zr_25_Ni_15_Cu_20_Sn_5_ at 930 °C/10 min.
