# Supplementary material for: Brazing of TC4 Alloy Using Ti-Zr-Ni-Cu-Sn Amorphous Braze Fillers
Source: Materials (Basel). 2024 Jul 29;17(15):3745. doi: 10.3390/ma17153745 (PMC11313286; doi:10.3390/ma17153745)
Supplement: Supplementary file 1 [file materials-17-03745-s001.zip › materials-3095156-supplementary.pdf]

Table S1. Element content (at.%) of each phase of the joint using $Ti_{35}Zr_{25}Ni_{15}Cu_{20}Sn_5$ at 930 °C/10min

Elements	Ti	Al	V	Zr	Ni	Cu	Sn	Possible Phase
E	79.55	8.35	3.21	4.33	2.09	1.40	1.07	α-Ti(s,s)
F	71.11	6.38	2.49	10.44	3.10	5.16	2.34	α-Ti(s,s) + $(Ti,Zr)_2(Ni,Cu)$
G	38.97	9.17	1.41	21.62	14.97	13.49	0.36	$(Ti,Zr)_2(Ni,Cu)$

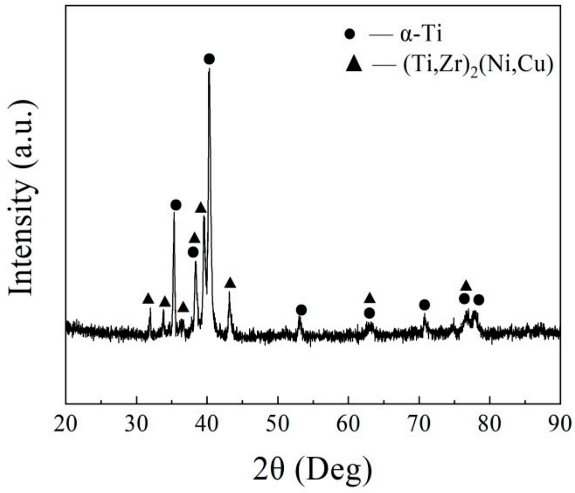

Figure S1. The XRD pattern of brazed joints using $Ti_{35}Zr_{25}Ni_{15}Cu_{20}$Sn5 at 930℃/10 min
